# Weakly supervised temporal model for prediction of breast cancer distant recurrence

**DOI:** 10.1038/s41598-021-89033-6

**Published:** 2021-05-04

**Authors:** Josh Sanyal, Amara Tariq, Allison W. Kurian, Daniel Rubin, Imon Banerjee

**Affiliations:** 1 Department of Biomedical Data Science, Stanford University School of Medicine, Stanford, USA; 2 Department of Biomedical Informatics, Emory University School of Medicine, Atlanta, Georgia; 3 Departments of Medicine and of Epidemiology & Population Health, Stanford University School of Medicine, Stanford, USA

**Keywords:** Breast cancer, Breast cancer

## Abstract

Efficient prediction of cancer recurrence in advance may help to recruit high risk breast cancer patients for clinical trial on-time and can guide a proper treatment plan. Several machine learning approaches have been developed for recurrence prediction in previous studies, but most of them use only structured electronic health records and only a small training dataset, with limited success in clinical application. While free-text clinic notes may offer the greatest nuance and detail about a patient’s clinical status, they are largely excluded in previous predictive models due to the increase in processing complexity and need for a complex modeling framework. In this study, we developed a weak-supervision framework for breast cancer recurrence prediction in which we trained a deep learning model on a large sample of free-text clinic notes by utilizing a combination of manually curated labels and NLP-generated non-perfect recurrence labels. The model was trained jointly on manually curated data from 670 patients and NLP-curated data of 8062 patients. It was validated on manually annotated data from 224 patients with recurrence and achieved 0.94 AUROC. This weak supervision approach allowed us to learn from a larger dataset using imperfect labels and ultimately provided greater accuracy compared to a smaller hand-curated dataset, with less manual effort invested in curation.

## Introduction

According to the World Health Organization, breast cancer is the fifth leading cause of cancer mortality worldwide, and breast-cancer-related mortality is mainly caused by metastasis and recurrence^1^. Recurrent breast cancer occurs months or years after the initial treatment. The cancer may come back on the same side of the breast as the original cancer (local recurrence), or it may spread to other areas of the body beyond the breast such as the bones, liver, lungs or brain (distant recurrence). Early prediction of breast cancer recurrence may help to guide a proper treatment plan. For example, patients whose chance of recurrence is very high may be considered for clinical trials of novel therapies to reduce metastatic recurrence, while those with lower probability may benefit from trials of treatment de-escalation^2,3^. Such predictions could also inform patients about their future risks, which may guide their life decisions.

With the advent of new digital technologies in medicine, large amounts of breast cancer data have been collected and are available to the medical research community^4–6^. Leveraging existing digital datasets, several predictive models have been proposed to aid breast cancer diagnosis and treatment^7–9^. However, the accurate prediction of a disease outcome is one of the most interesting and challenging tasks since it can help to discover and identify patterns and relationships between the complex electronic healthcare datasets and effectively predict future outcomes which will contribute to individualizing cancer treatment.

Recurrence prediction models that have been published to date^10–12^ used only a limited dataset for training and validation which may result in a highly simplified and conservative model with less generalizability for complex cases. The reason for using such a small dataset is mainly motivated by the fact that obtaining manually curated recurrence labels needs through chart-review by content experts, which is costly and impractical for more than 1000 cases. In such a situation, weak supervision is becoming a popular strategy in the machine learning field where noisy, limited, or imprecise sources are used for labeling large amounts of training data.

Natural Language Processing (NLP) models have been developed to extract breast cancer recurrence information from clinical notes, electronic health records (EHR) and population-based cancer registries, with varying degrees of success in generating labels. Such NLP-generated labels can be used for weak supervision of a breast cancer prediction model, letting the model leverage a large-scale EHR for training. Carrel et al.^13^ were able to extract information from clinical notes and identify 92% of recurrent breast cancer cases. Soyasal et al.^14^ used NLP to extract information about metastatic sites for lung cancer patients and were able to achieve recall of 0.84 and 0.88 precision for metastatic status detection, and 89% recall and 93% precision for metastasis site detection. In our previous work, we developed a neural network-based NLP approach to extract breast cancer recurrence timeline information from progress notes, radiology and pathology reports and were able to achieve a sensitivity of 83%, specificity of 73% and AUROC of 0.9 for recurrence detection^15^.

In the current study, we propose a weak-supervision framework to predict breast cancer recurrence one year in advance using only unstructured clinical narratives, including progress notes, pathology and radiology reports, and nursing notes. The weak-supervision leverages our previously developed NLP model and allows us to generate a large annotated breast cancer recurrence dataset (8,956 breast cancer patient treated in Stanford between 2000 to 2018) for training the prediction model which to our knowledge is the first model to learn on such huge dataset. To evaluate the weak-supervision framework performance, we compared performance of two approaches—(1) weak-supervision #1—trained with manually curated data and our NLP-generated labels with optimal sensitivity and specificity and (2) weak-supervision #2—trained with manually-curated data and the NLP-generated labels with high sensitivity (only few false negative cases which ultimately increase the number of positive cases), against a traditional prediction model (only trained with manually curated data).

## Results

We evaluate our research pipeline by broadly categorizing them into two components—(1) the semantic quality of the words and clinical notes embedding generated by the NLP methods through visualizations, (2) the performance of the temporal LSTM model to predict recurrence where we assess the performance of our model under different weak-supervision settings.

### Textual embedding evaluation

**Figure 1:**
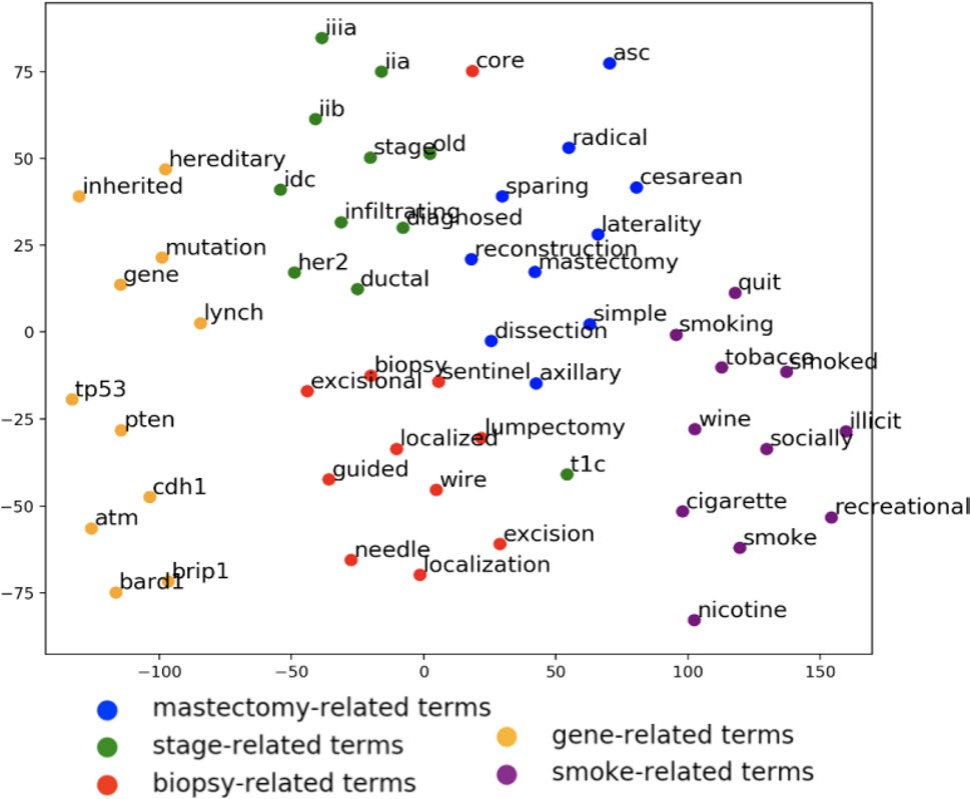
2D visualization of word embeddings computed using t-SNE^16^.

We evaluated the semantic quality of the embedding generated by our vectorization pipeline on two different levels. On the word-level, we found similar word clusters in a totally unsupervised manner to verify the positioning of synonyms (and related words). This suggests that our vector embedding is able to preserve legitimate semantics of the natural words and clinical terms. We first selected five clinically significant terms (mastectomy, stage, biopsy, gene, smoking) and for each of these, their ten most similar words. To determine the similarity between words, we calculated the cosine distance between the two-word vectors, with higher cosine values indicating higher similarity. Next, we used t-SNE^16^ to reduce the dimensionality of the 300 length word vectors down to 2-dimensional space for visualization as shown in Fig. 1. The t-SNE dimensionality reduction technique was chosen because it reduces the tendency to crowd points in the center of the space, making it easier to visualize the separate clusters formed after training the word embeddings.

**Figure 2:**
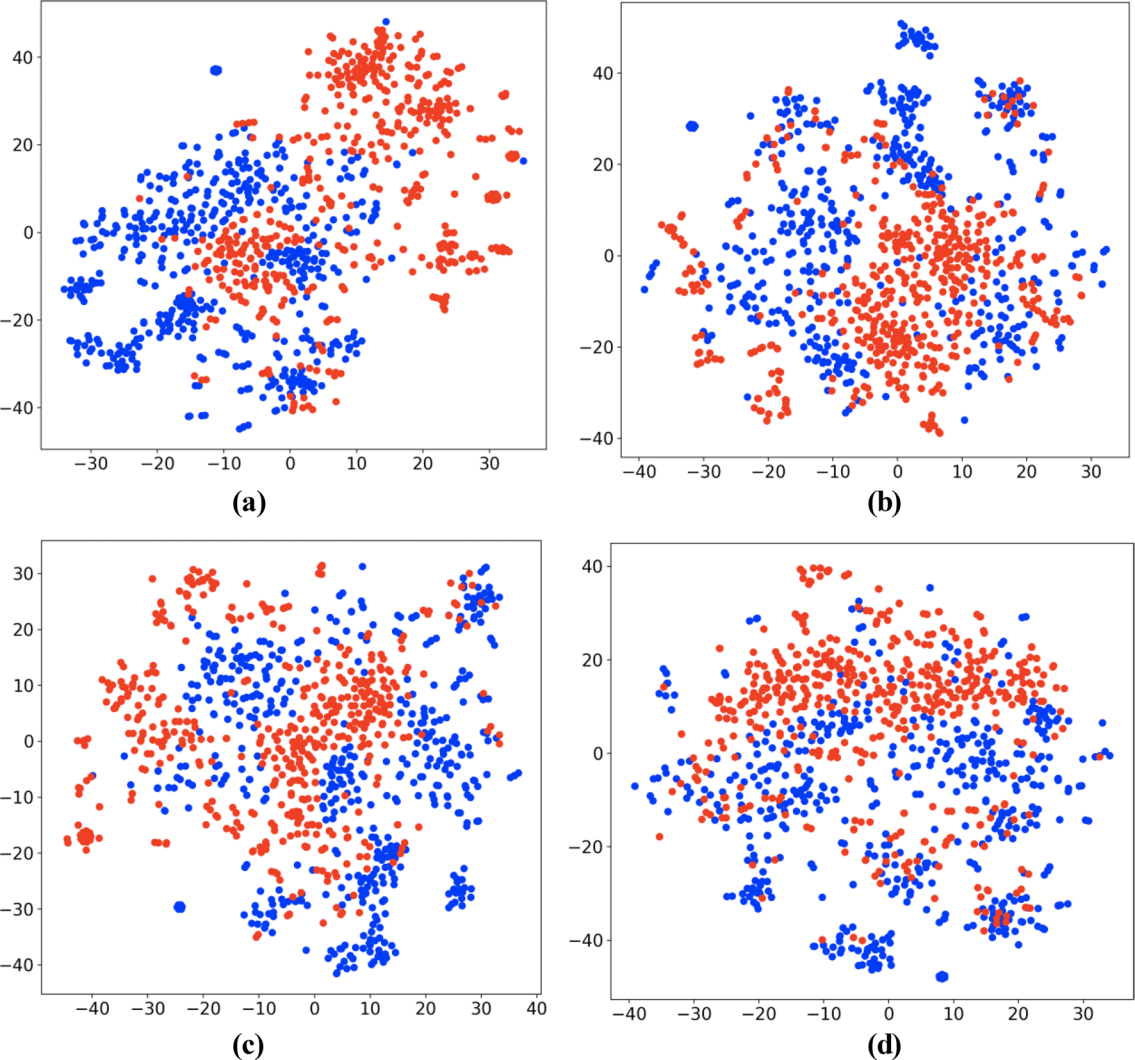
T-SNE visualization of document vectors labeled at (**a**) recurrence within 0 months, (**b**) recurrence within 3 months, (**c**) recurrence within 6 months, (d) recurrence within 1 year.

At the note-level, we visualized the document vectors of 1000 notes, with 500 randomly selected from each class to fulfill the purpose of analyzing the proximity of documents that have different recurrence labels. We labeled each document using the patient’s recurrence status at the time of the note’s creation (Fig. 2a), 3 months in the future (Fig. 2b), 6 months in the future (Fig. 2c), and 1 year in the future (Fig. 2d), with recurrence labeled as red and no recurrence as blue. If the documents corresponding to the same class (risk) appeared close to each other and form clusters, we inferred that our embedding carried substantial signals for recurrence, which could be useful to boost the performance of any standard classifiers. As we can see from the t-SNE projection in Fig. 2a, recurrence-positive cases (red) and negative cases (blue) formulate natural clusters which show that the vectorization was able to preserve the semantics of the clinical notes. Even as the labels represent timepoints further in the future, the subsequent visualizations still contain natural clusters, indicating that the document vectors, alone, are informative for the task of recurrence prediction.

### LSTM prediction evaluation

**Figure 3:**
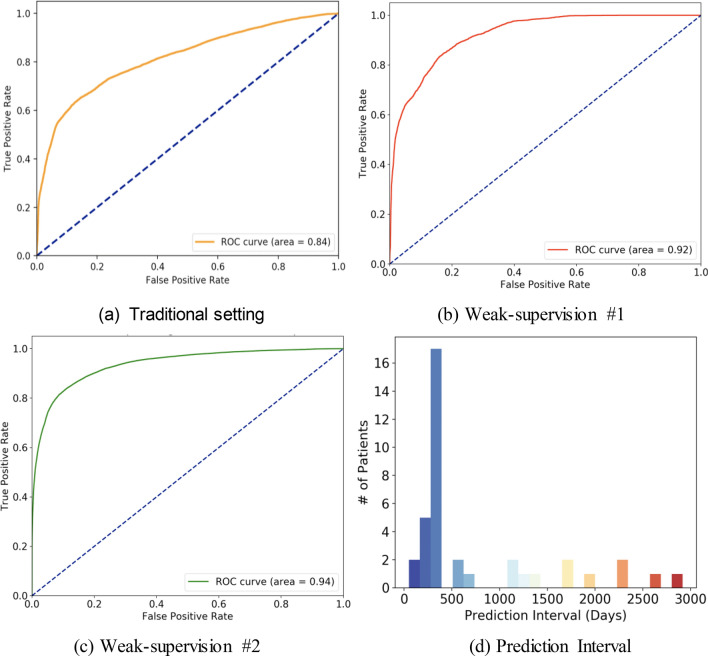
LTSM model performance evaluated as Receiver Operating Characteristic curves trained with (**a**) 670 manually curated patients, (**b**) weak supervision combining manually curated (670 patients) and NLP generated labels of 7437 patients, (**c**) weak supervision combining manually curated (670 patients) and NLP generated labels of 7437 patients with more positives samples. (**d**) The distribution of prediction interval (time between positive recurrence prediction and actual recurrence) for patients in the test cohort using the best performing LSTM model.

Figure 3 shows the model performance for distance recurrence prediction (1 year advance) in three distinct settings on the same annotated test cohort containing 224 patients where the recurrence timeline has been identified by manual chart-review. The optimal prediction performance was 0.94 ROC AUC and at the optimal operating point of ROC, the sensitivity was 0.89 and specificity was 0.84. This was achieved in weak-supervision #2 setting where the manually curated data were combined with NLP-generated labels with high sensitivity in order to leverage more positive samples in training. In weak-supervision #1 setting, the model obtains 0.92 ROC AUC, and 0.83 Sensitivity, 0.85 Specificity. This drop-in performance could be explained by the lower number of positive recurrence cases available in training for weak-supervision #1. While the lowest prediction performance was obtained in the *traditional setting* with all manually curated training data*,* the model achieved 0.84 ROC AUC, and at the optimal operating point of the ROC, the sensitivity was 0.72 and specificity was 0.82.

To better characterize the timeline over which the model is able to predict recurrence before the event, we create a histogram in Fig. 3.d to illustrate how far in advance the model captures the positive cases of recurrence in the test cohort. This is done using the best performing LSTM model and for each patient, we determine the earliest date when the model predicts a recurrence probability that exceeds the threshold set by the optimal operating point and find the difference between then and the confirmed recurrence date. The majority of successfully predicted patients (57.9%) are predicted positively for recurrence within 300–400 days of the actual recurrence date, indicating the model’s ability to successfully capture cases of recurrence at the intended time.

To evaluate the extent to which the LSTM architecture is improving performance by incorporating information across a large timeline of notes, we compare the performance of the LSTM model to a traditional machine learning model trained to predict recurrence using a single vectorized note. We choose XGBoost^17^, an implementation of gradient-boosted decision trees, for this comparison as it has achieved state-of-the-art results on a variety of machine learning tasks. To ensure comparable results, this XGBoost model was trained using the same 3 supervision strategies and evaluated on the same test cohort. As shown in Table 1, the XGBoost model achieved its best performance in weak supervision #2 with 0.81 ROC AUC, 0.77 sensitivity, and 0.73 specificity. The significant drop in performance between this model and the LSTM model across the different supervision strategies indicates that the LSTM architecture is able to capture complex temporal relationships in the note vectors beyond the information provided innote vectors at individual time points. Furthermore, the LSTM architecture is better able to improve its performance through the addition of weakly labelled data when compared to the performance boost from weak supervision in the XGBoost model.

**Table 1:** Performance of the temporal LSTM and XGBoost models compared across all three supervision strategies.

	Temporal LSTM model			XGBoost		
	ROC AUC	Sensitivity	Specificity	ROC AUC	Sensitivity	Specificity
Traditional supervision	0.84 ± 0.07	0.72 ± 0.07	0.82 ± 0.05	0.76 ± 0.10	0.67 ± 0.09	0.72 ± 0.07
Weak supervision #1	0.92 ± 0.05	0.83 ± 0.08	0.85 ± 0.06	0.79 ± 0.08	0.71 ± 0.08	0.73 ± 0.07
Weak supervision #2	0.94 ± 0.04	0.89 ± 0.07	0.84 ± 0.06	0.81 ± 0.07	0.77 ± 0.06	0.73 ± 0.08

**Figure 4:**
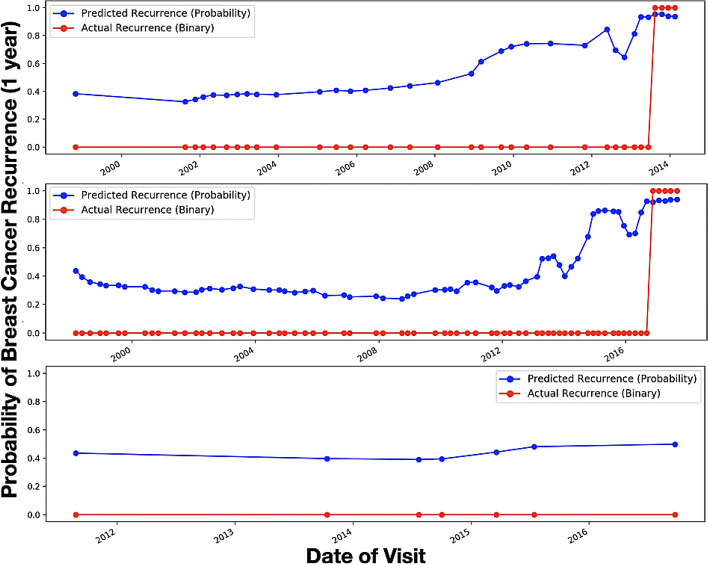
Predicted recurrence probability plotted against the actual recurrence status over time for three separate patients—x-axis shows the encounter date in ascending order starting from the first visit, y-axis shows the probability. Red line represents the ground truth: Definite recurrence = 1, No Recurrence = 0. Blue line represents the predicted probability score.

The overall high accuracy of the recurrence prediction model with weak supervision suggests that the proposed model performed quite well on estimating recurrence one year before it occurred on the test set. In Fig. 4, we present the graph summary for multiple randomly selected patients which shows that the predicted probability sequence closely follows the ground truth labels.

## Discussion

Prediction of breast cancer recurrence from clinical notes is a challenging problem; it not only needs an efficient way to handle ambiguity in the clinical text but also requires a sequential prediction model to capture the complex correlation between information from current and historic encounters. We propose a sequential deep learning model using LSTM which reads the clinic notes in order, creates note-level embedding and predicts recurrence risk one year in advance. The model was trained using a NLP-generated, large, labeled breast cancer dataset.

The core contribution of this work is (1) development of a robust and efficient breast cancer recurrence prediction model using weak supervision; (2) embedding of clinical notes using weighted vectorization. Although in the current deep learning field, weak-supervision frameworks are very popular, application of weak supervision for clinical prediction could be perceived in a different way from the generic application. In clinical prediction, limited representation of the relevant class (often only 1–5% of the overall dataset) is the greatest challenge, even more than a lack of annotated samples. Thus, we proposed three parallel strategies for recurrence prediction—(i) *traditional:* train with limited manually curated data, (ii) *weak supervision 1:* train with weakly labeled data with high specificity (only few false recurrence—less positive cases), and (iii) *weak supervision 2:* train with weakly labeled data with high sensitivity (only few false no recurrence—more positive cases). We showed that our weak supervision model with more positive cases obtained optimal prediction performance with 0.94 ROC AUC, 0.89 sensitivity and 0.84 specificity, and outperformed not only the model trained with manually curated data but also the weak supervision model with fewer positive cases.

Previous studies^10–12^ applied machine learning technology to predict breast cancer recurrence using demographics, procedure codes, diagnosis, treatment, prescription filling, chemotherapy codes, pathological, and genetic data. However, most of the previous breast cancer recurrence prediction models were either developed using only structured clinical data fields or with linear classification models that had limited success to model the complex co-relations of sparse structured clinical data^18^. For example, Nordstrom et al. selected a limited subset of the structured indicators for breast, non-small cell lung and colorectal cancer, used a traditional Random Forest classifier to predict recurrence, and concluded that their algorithm performed at approximately the same level as the presence of a secondary tumor diagnosis code, with low sensitivity and high specificity^19^. Lamont et al.^20^ also used only structured information from Medicare and Medicaid data and Chawla et al.^21^ used SEER cancer registries and Medicare claims data to identify metastasis. Clinical notes, including progress notes, radiology and pathology reports, are documented mostly in a free-text format but may offer the greatest nuance and detail about a patient’s clinical status. However, clinic notes are mostly unexplored or under-explored with a simplistic processing approach for recurrence prediction due to the challenge of representing the unstructured nature of free-text data including varying structures, semantic qualities, and size, in a computer-manageable way. Some interesting work has been done in the area of cancer prediction with structured and unstructured data. Zhao et. al.^22^ used a key-word based search and Bayesian inference combining EHR and Pubmed abstracts for pancreatic cancer prediction.

We leveraged our previously published NLP methodology to curate a large set of training data for metastatic recurrence of breast cancer. This approach alleviates the expense of manual chart-review and can be used to create a strong predictive model trained on a large patient cohort. Our experiments showed that a weak-supervision framework with NLP generated labels with high sensitivity and enabled the model to learn from a large dataset, ultimately improving its performance on the evaluation set. The proposed weighted vectorization scheme for clinical notes also preserved clinical semantics, which ultimately boosted the model’s prediction performance. Our proposed framework will also be transferable to other clinical event prediction tasks with minimal fine-tuning. Core limitation of our work is that the model was trained using single institutional data that contains biases regarding syntactic style of clinical narratives, patient populations, and treatment planning. In future, we plan to include multi-institutional test dataset to validate our model.

## Methods

**Figure 5:**
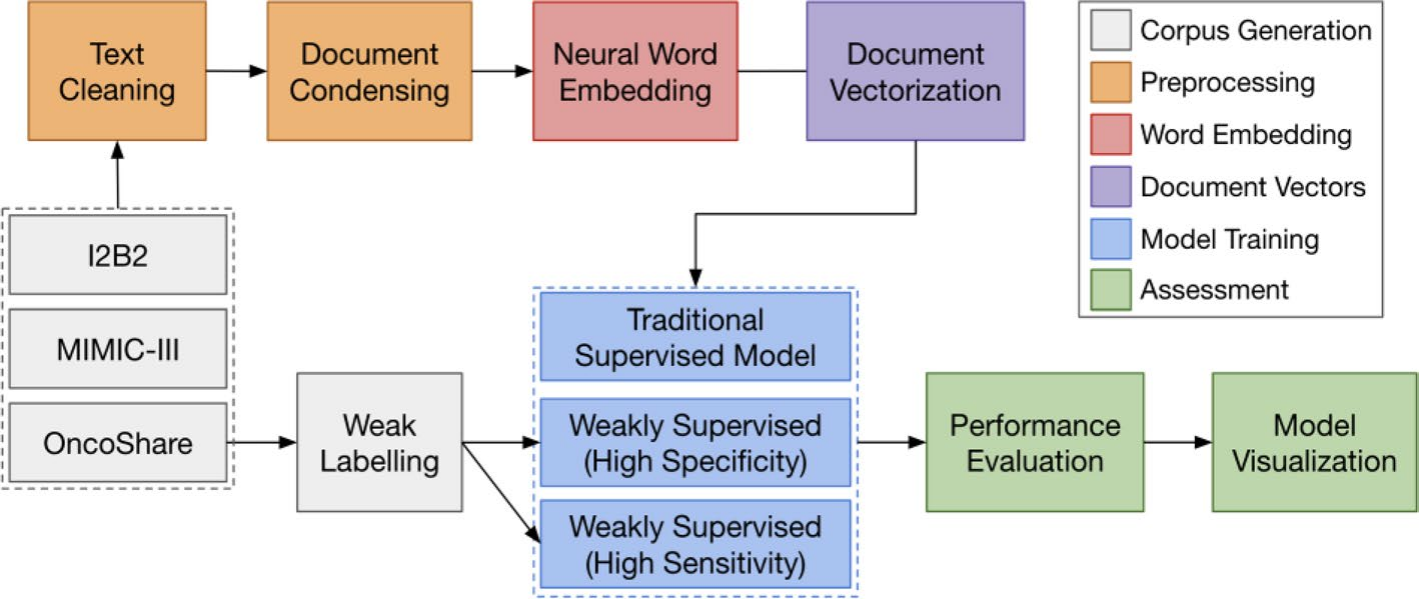
Proposed Research Workflow. The interaction between the components is shown by the arrow. Output from the previous component is being passed to the next component for analysis.

Figure 5 presents the proposed research workflow which can be broken down into core processing blocks: 1. *Text cleaning*—preprocessing of the clinical notes, 2. *Neural Embedding*—learn the language space, 3. *Document vectorization*—vector representation of the notes, 4. *Prediction models*—training three prediction models, and 5. *Performance evaluation and Visualization*.

### Cohort generation

#### Patient cohorts

In this study, we used two publicly available clinical note datasets to curate a corpus of generic clinical notes and learn generalizable note representations. Oncoshare, a patient-specific corpus obtained from Stanford University, is then used to train the recurrence prediction model. Characteristics of these datasets are summarized in Table 2.

**Table 2:** A variety of datasets are used to train generable note representations—description and statistics.

Dataset	Size	# Notes	Location	Description	Mean # sentences	Mean # words
I2B2^23^	24.2 MB	2.2 K	Beth Israel deaconess medical center + partners	Smoking, heart disease, obesity, etc	75.21 (51.13)	1065.81 (689.19)
MIMIC-3^24^	4.01 GB	91.7 M	Beth Israel deaconess medical center	Intensive care unit visits	19.14 (24.43)	442.90 (823.59)
OncoShare^25^	2.79 GB	893 K	Stanford healthcare	Breast cancer recurrence	17.16 (37.43)	122.63 (387.54)

#### Generic cohort

To learn the clinical language space, we curated a generic clinical note cohort by compiling 92.6 million open-source clinical notes from the I2B2 NLP research database^23^ and MIMIC-III critical care database^24^. The I2B2 research datasets are composed of fully de-identified notes collected from 2004 to 2014 and are available for general research purposes. The MIMIC-III database contains the data of patients who stayed in the intensive care units at Beth Israel Deaconess Medical Center. Several tables track different patient variables such as diagnoses, lab events, and prescriptions, but we used only the note events table, which consists of de-identified notes including discharge summaries, ECG reports, and imaging reports.

#### Oncoshare cohort

**Figure 6:**
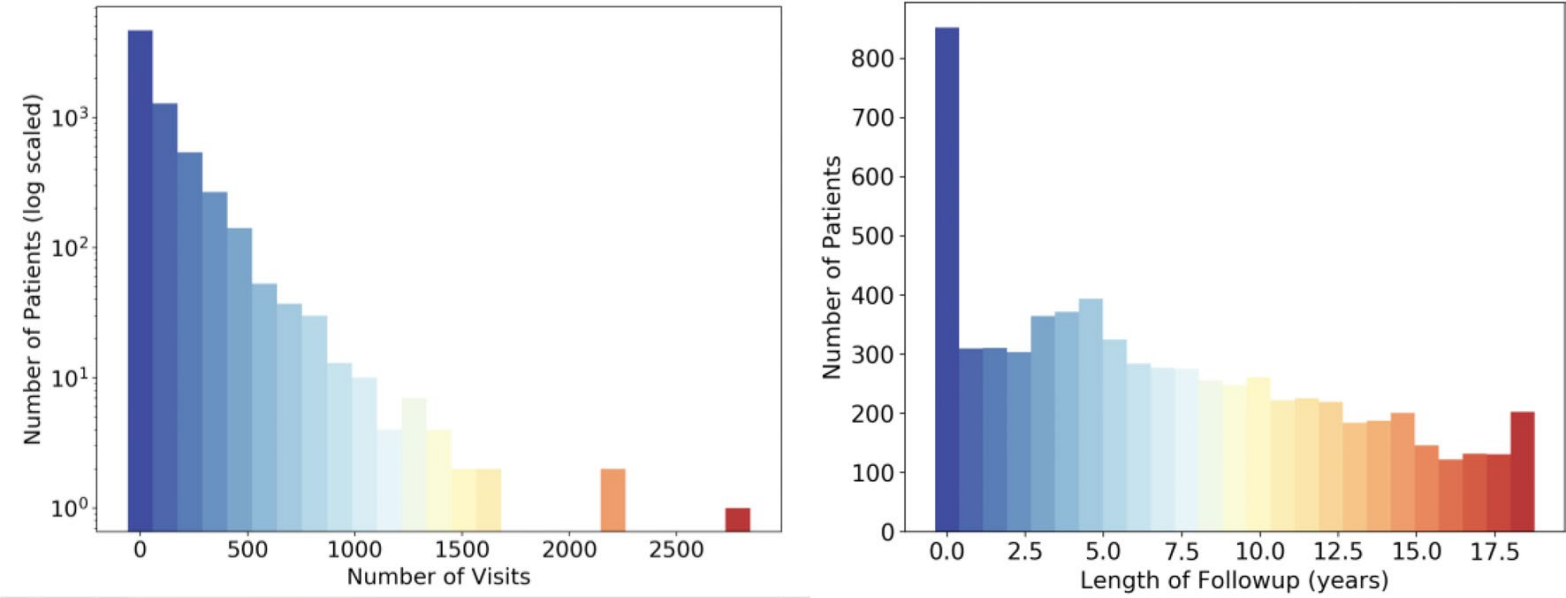
Distribution of visits (left) and follow-up times (right) for all patients in the breast cancer recurrence dataset.

With the approval of Stanford University Institutional Review Boards, we used the Oncoshare breast cancer database^25^ which was developed as an integration of EHRs of Stanford Health Care (SHC), an academic health institution, and multiple sites of the Palo Alto Medical Foundation (PAMF), a community-based medical center in Northern California, and the California Cancer Registry. Table 3 presents the characteristics of the Oncoshare cohort. Following the Stanford IRB regulation, informed consent was obtained from the patients. The database captures individual patient-level information such as diagnoses, procedures, prescriptions, and clinical notes. For this study, we focused on 892,550 free-text clinical notes (medical and social histories, impressions, visit summaries, etc.) collected from 8,956 de-identified breast cancer patients treated from 2000 to 2018 at SHC. Figure 6 illustrates the distribution of notes per patient in the Oncoshare dataset. The mean number of notes per patient is 126 with a standard deviation of 172 notes. On average, patients had 7.46 years of follow-up data in Oncoshare with a standard deviation of 5.48 years.

**Table 3:** Characteristics of the Oncoshare patients: overall and manually curated samples.

	Whole dataset (patients: 8956)	Manually Chart-reviewed (N = 1,519)
Age at primary diagnosis	54 (± 13)	54 (± 12)
Follow-up duration	6 years	5 years
**Marital status**		
Single	1354 (15%)	139 (9.15%)
Married	5869 (65%)	1048 (69%)
Separated/divorced/widowed	1539 (17%)	130 (8.55%)
Domestic partner	11 (0.1%)	0 (0%)
Unknown	183 (2%)	202 (13.29%)
**Ethnicity**		
Hispanic	842 (9%)	60 (4%)
Non-hispanic	7649 (85%)	1002 (66%)
Unknown	465 (5%)	457 (30%)
**Race**		
White	6726 (75%)	759 (50%)
Asian	1353 (15%)	212 (14%)
Black	325 (4%)	46 ( 3%)
Pacific Islander	49 (1%)	18 (1%)
Native American	17 (1%)	2 ( 0%)
Unknown	486 (5%)	482 (32%)
**Stage from Stanford cancer registry**		
0	419 (5%)	61 (4%)
1	1041 (12%)	182 (12%)
2	23 (1%)	1 (0%)
3	441 (5%)	133 (8%)
4	53 (1%)	0 (0%)
Unknown	6979 (78%)	1142 (75%)
**Payer**		
Not insured	61 (1%)	15 (1%)
Insurance, NOS	891 (10%)	121 (8%)
Managed care/Health Management Organization/Preferred Provider Organization	299 (3%)	7 (0%)
Medicaid	643 (7%)	91 (6%)
Medicare	1643 (18%)	212 (14%)
Others	347 (4%)	60 (4%)
Unknown	5072 (57%)	536 (69%)
**Grade**		
Grade 1 (Well differentiated)	1609 (18%)	273 (18%)
Grade 2 (Moderately differentiated)	3363 (38%)	531 (35%)
Grade 3–4 (Poorly differentiated)	3984 (44%)	713 (47%)

#### Recurrence labels for the Oncoshare data

All experimental protocols were approved by a Stanford University Institutional Review Boards and all methods were carried out in accordance with relevant guidelines and regulations. Among the 8,956 patients in the Oncoshare database, we selected 1,519 patients to perform manual chart-review to establish whether patients had distant metastatic recurrence (designated as “definite recurrence” or “no recurrence”). We presented the patient characteristics in Table 3. For manual chart-review, we recruited three senior medical students to undertake a chart review of each patient using a Web-based in-house tool^26^. Subsequently, two senior oncologists removed the uncertain patients and finally, 894 patients served as the ground truth data set. The mean inter-annotator agreement was 0.81. More detail about the annotation can be found in our previous publication^15^. The NLP method scored 0.9 AUROC for detecting the recurrence timeline of the test data (quarter-wise) and at the optimal operating point, the method obtained 0.82 specificity and 0.73 sensitivity. The patient-level performance was 0.95 specificity and 0.93 sensitivity. All the remaining patients’ notes in the Oncoshare database (7437 patients) were weakly labeled by the neural network-based probabilistic NLP method. Using the probabilistic classifications, we used two different thresholds to separate them into positive and negative recurrence cases, the higher threshold focused on capturing only a few recurrences with high specificity, and the lower threshold focused on detecting more recurrences with high sensitivity. Specifically, the higher threshold resulted in 19,766 notes with positive recurrence status while the lower threshold resulted in 165,630 notes with positive recurrence status.

### Text cleaning: preprocessing of the clinical notes

**Figure 7:**
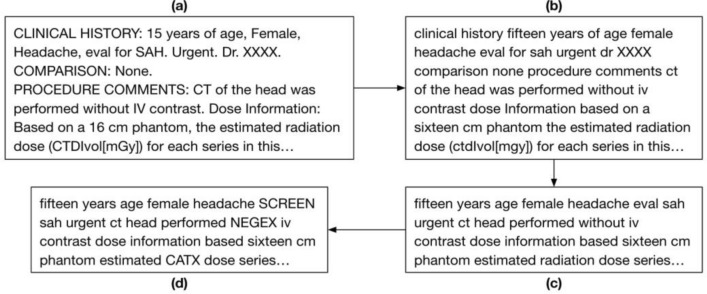
Pictorial representation of a free-text radiology report (**a**), transformed through the preprocessing steps of text cleaning (**b**), unwanted phrase removal (**c**), and word mapping (**d**).

To reduce the linguistic and style variability, each note is transformed through a series of pre-processing steps as illustrated by Fig. 7. We first processed the notes using standard text cleaning steps—by removing excess whitespace and punctuation, converting all text to lowercase letters, and converting all numbers to words. Afterwards, we remove the following unwanted terms and phrases to eliminate noise: general stop words, words with less than 50 occurrences in the corpus, general section headings (“impression”, “findings”), medico-legal phrases, and proper nouns. These words provide little value in predictions and increase computation time when training word embeddings. Following this step, we use the publicly available CLEVER terminology (https://github.com/stamang/CLEVER/blob/master/res/dicts/base/clever_base_terminology.txt) to map similar common terms and related domain-specific terms to controlled terms. For example, common terms like “brother”, “mother”, and “son” were mapped to “FAM” and domain-specific terms such as “cancer”, “lesion’, and “oncology” were mapped to “CA”.

### Neural embedding: learn the language space

In order to represent the words appearing in the clinical notes into a machine-understandable form, we created a Language Space Model that generates embedding of the words in a vector space where vector representation of similar words are mapped near each other. We trained a language space following a distributional semantics approach where we used all the preprocessed text corpus (I2B2, Mimic-III, Oncoshare) and learned in an unsupervised manner. We used the word2vec model, introduced by Mikolov et al^27^. The word2vec model iteratively analyzes context words around a specific center word to learn memory-efficient vector representations of each word while keeping the word similarity based on context. We use the skip-gram variant of word2vec which aims to predict these context words from the center word as opposed to continuous bag-of-words (CBOW) which does the inverse by predicting the center word given context words. While CBOW is faster, we use skip-gram because it performs better for infrequent words which may be informative for recurrence prediction, given recurrence is minority class. This was confirmed in cross validation on the training data, where optimal performance was achieved by a skip-gram architecture with a vector size of 300 and window size of 30. All other parameters were set to their default settings and the model was trained for 30 epochs. The model is trained on a total 92.6 million clinical notes.

### Document vectorization: weighted vectorization of the notes

We create note-level embeddings for the 892,550 clinical notes by computing a weighted average of all word vectors in each clinical note. First, we calculate the tf-idf (term frequency-inverse document frequency) score for all unique words in each note as: $tf*\mathrm{log}(\frac{N}{df})$, where *tf* is the number of occurrences of the word in the note, *df* is the number of notes with the word, and *N* is the total number of notes. This score is high for terms which occurs many times in a note, but infrequently throughout other notes in the corpus. These scores are then used as a weighting factor for each word vector, with high tf-idf scores emphasizing potentially clinically significant and discriminative features which are informative from recurrence prediction. Finally, each note is represented as an average of all of these word vectors weighted by their tf-idf score as: ${V}_{note}=\frac{1}{N}\sum_{i=1}^{N}{W}_{i}*{V}_{{W}_{i}}$, where ${V}_{{W}_{i}}$ is the vector representation of *i*-th word, ${W}_{i}$ is tf-idf weight, and $N$ is the total number of words in the preprocessed note. Initial experiments showed that this unsupervised approach performed better than other document representation techniques such as doc2vec, TF-IDF, and a simple average of word2vec.

### Recurrence prediction model

**Figure 8:**
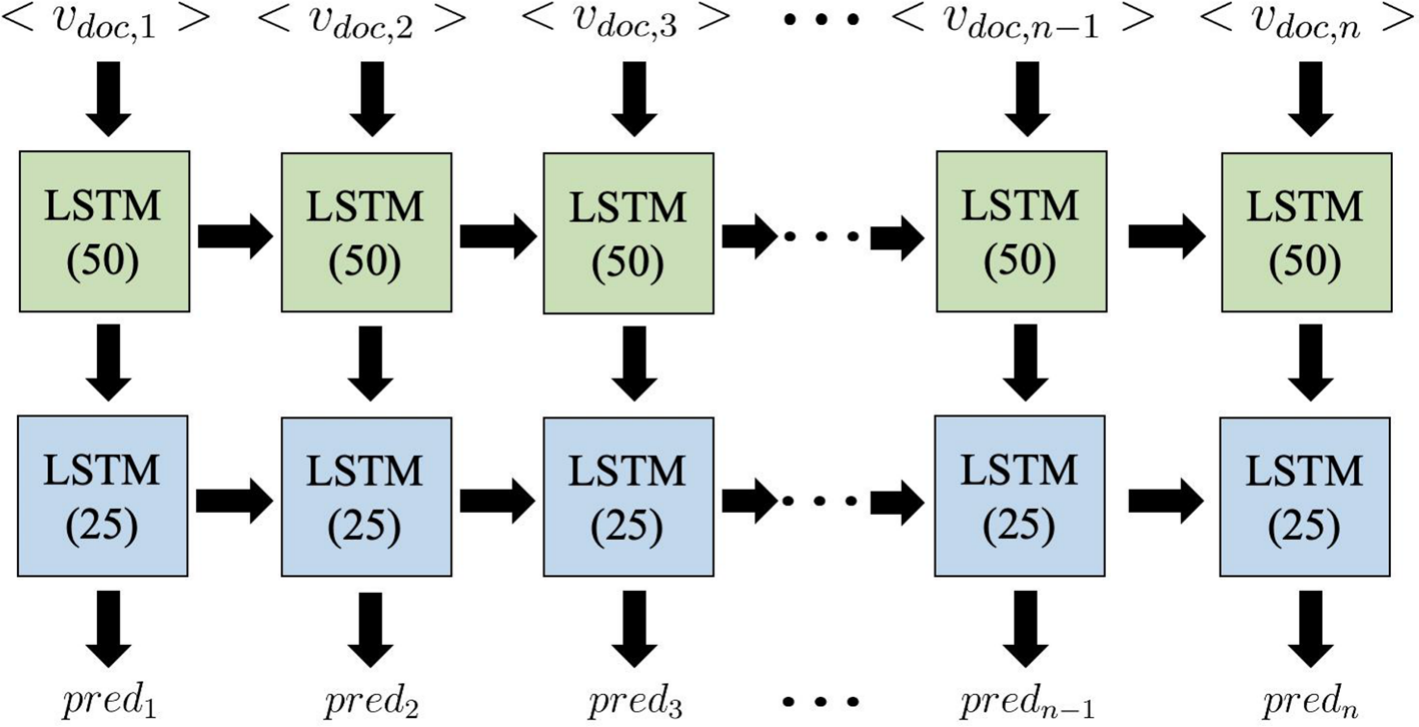
LSTM model architecture for breast cancer recurrence prediction.

Using the note-level embeddings, we train a recurrent neural network (RNN) with long short-term memory (LSTM) units^28^ to predict the probability of breast cancer recurrence in 1 year. This model architecture is designed to process each patient’s clinical note ordered based on encounter timestamp as a longitudinal series over time. We chose to use LSTM units over vanilla RNN units based on the fact that LSTM better retains memory of important events in a long time series. The long-term memory encodes general information regarding the entire visit sequence while short-term memory keeps track of immediate changes in patient visits. The model architecture as displayed in Fig. 8 consists of a 1-directional, many-to-many, stacked stateful LSTM with 2 layers and a total of 78 K trainable parameters. The first layer consisted of 50 hidden neurons, followed by batch normalization and 20% dropout. The next LSTM layer consisted of 25 hidden neurons, followed by 20% dropout. Batch normalization and dropout are used to accelerate training, prevent overfitting, and improve overall model performance.

Prior to training, the notes of each patient are concatenated in chronological order and this sequence of notes can be represented as ${X}_{i}={({V}_{i}}^{(1)},{{V}_{i}}^{(2)},\ldots,{{V}_{i}}^{(n)})$ where ${{V}_{i}}^{(t)}$ represents the vector representation of the i-th patient’s t-th note. To preserve as much meaningful data without vastly increasing computational time, we set the maximum length of these sequences to 800 notes based on the distribution of visits for all patients as shown in Fig. 6. While only 79 patients had more than 800 notes, there were three patients with over 2000 notes (2291, 2310, 2900) and using all of these notes would result in more padded data than actual visits. Removing all notes beyond each patient’s 800th note dramatically reduced computational time and only eliminated 2.6% (23,237) of all notes. For patients with less than 800 notes, their note sequences are padded to 800 length using the zero vector. These sequences of notes are then labelled as ${Y}_{i}={({y}_{i}}^{(1)},{{y}_{i}}^{(2)},\ldots,{{y}_{i}}^{(n)})$ where ${{y}_{i}}^{(t)}$ represents the recurrence status of the patient 1 year from the note’s date and padded notes are assigned a separate class label.

The one directional LSTM units aim to predict recurrence probability by using the notes from all current and previous time points. The first-layer LSTM unit at time point *t* receives the input of ${{V}_{i}}^{(t)}$ which represents the current note and the previous unit’s hidden state ${h}^{(t-1)}$ which captures the relevance of all prior notes. This unit then passes the current hidden state ${h}^{(t)}$ to its corresponding second-layer unit and the successive first-layer LSTM unit. The second-layer LSTM units at time point *t* then use these hidden states and recurrent connections with previous second-layer units to compute ${\widehat{y}}^{(t)}=softmax(L\circ {h}^{(t)})$, a vector of three probabilities which correspond to no recurrence, positive recurrence, and padded note. The trainable parameters for each LSTM block in the model include the input-to-hidden matrix in layer 1, hidden-hidden matrix in layer 2, and the bias vector.

We optimized the model using weighted Categorical Cross Entropy loss and used the Adam optimizer^29^ with a decaying learning rate starting at 10^–3^. During the loss estimation, the padded notes were assigned zero weight to ensure they do not affect model training. In total, the model was trained for 20 epochs with a batch size of 32.

Using the same prediction model architecture and hyperparameters, we compare the performance of the traditional model trained on the manually curated labeled data against two weakly supervised approaches which incorporate the remaining 8,062 patients annotated by the NLP algorithm. In order to evaluate the performance using weakly supervision, the test cohort of 224 patients fixed across all three models to ensure comparable results.

### Performance evaluation

The manually labeled set of 894 patients was randomly split into a 75% training set (670 patients) and 25% test set (224 patients). To optimize hyperparameters such as learning rate and the number of hidden neurons, the model was validated on the training set via fivefold cross validation. Following validation, the model was trained on the entire training set and performance was computed using the holdout test set, treating each note as an individual sample for evaluation. We also exclude all notes from the test set that occur after the date of recurrence to ensure we are measuring the model’s predictive ability rather than detection. We choose to measure the performance using the area under the receiver operating curve (ROC AUC) based on its ability to accurately determine model performance from 1 (perfect) to 0, despite class imbalance or a specific threshold. This allows clinicians to choose their preferred tradeoff between specificity and sensitivity. In addition to this primary metric, we selected the operating point that maximizes the sensitivity and specificity to determine a potential model threshold and report the model’s sensitivity and specificity as additional metrics for performance measure.

## Data Availability

The datasets generated during and/or analysed during the current study are not publicly available due to the patient data privacy restriction, but de-identified subset of data is available from the corresponding author on reasonable request. The prediction models can be obtained through an MTA.

## Code availability

Authors are committed to release the code in the Dr. Banerjee’s GitHub respository for public access with open source licensing.
